# The Effect of Choline Salt Addition to Trehalose Solution for Long-Term Storage of Dried and Viable Nuclei from Fully Grown Oocytes

**DOI:** 10.3390/bioengineering10091000

**Published:** 2023-08-24

**Authors:** Joseph A. Orozco Cabral, Pei-Chih Lee, Shangping Wang, Yizhou Wang, Yong Zhang, Pierre Comizzoli, Gloria D. Elliott

**Affiliations:** 1 Department of Mechanical Engineering and Engineering Sciences, University of North Carolina at Charlotte, Charlotte, NC 28223, USA; jandorocab@gmail.com; 2 Smithsonian’s National Zoo and Conservation Biology Institute, Washington, DC 20008, USA; leep@si.edu; 3 Department of Bioengineering, Clemson University, Clemson, SC 29634, USA; shangpw@clemson.edu; 4 Nanoscale Science Graduate Program, University of North Carolina at Charlotte at Charlotte, Charlotte, NC 28223, USA; ywang137@charlotte.edu; 5 Electrical and Computer Engineering Department, University of North Carolina at Charlotte, Charlotte, NC 28223, USA; yong.zhang@charlotte.edu

**Keywords:** microwave-assisted dehydration, oocyte nucleus, trehalose, choline acetate, relative humidity

## Abstract

Although drying techniques are exciting alternatives to cryopreservation, it remains challenging to maintain tightly controlled temperatures and humidity levels during storage of dried products. The objective of this study was to determine if the addition of choline acetate to trehalose solution could enable a wider range of storage conditions for preservation of nuclei from fully grown oocytes, by allowing temporary humidity excursions (>44% relative humidity) that may lead to crystallization of trehalose and loss of DNA integrity. Using domestic cat germinal vesicle oocytes as a model, we characterized the recovery as well as the integrity of samples after microwave-assisted dehydration. Exposure to choline acetate alone did not impair the germinal vesicle’s DNA integrity and only had a negative impact on the chromatin configuration. Choline acetate addition enabled us to reach lower moisture contents after 25 min of microwave-assisted drying. Sample recovery after rehydration was also better in the presence of choline acetate. The integrity of the germinal vesicle’s DNA was not affected, while the chromatin configuration was impaired by the presence of choline acetate during dehydration. Importantly, choline acetate addition helped to maintain an amorphous state (absence of detrimental crystallization) during excursion from ideal humidity conditions.

## 1. Introduction

Preservation of sperm and oocytes at freezing temperatures has revolutionized the reproductive biology and fertility fields in human and animal species [1,2]. However, compared to the success and extensive use of sperm cryopreservation, oocytes remain more challenging to preserve and store for the long term [2,3]. Cryopreservation involves the reduction of the temperature of a tissue or cell down to an extremely low level to halt biological processes and stop molecular mobility [4]. To prevent damaging ice crystals from forming inside cells during cooling, the addition of a cryoprotective agent is necessary [4,5]. While small cells can be cryopreserved quite effectively using moderate levels of cryoprotectants, the high ratio of volume to surface area in large cells, such as oocytes, makes it difficult to load adequate amounts of cryoprotectant without toxicity concerns [6]. Furthermore, oocytes often do not dehydrate adequately during slow cooling, resulting in trapped water that can form damaging intracellular ice at low temperatures. Ultra-rapid freezing or ice-free vitrification methods have been developed to mitigate these issues, but these methods require higher levels of toxic cryoprotectants, and can only be applied to small numbers of cells at a time to attain the rapid cooling rates required [2]. Lastly, cryopreservation and biobanking are costly and technically intense as they require liquid nitrogen supplies, adapted facilities, and alarm systems [2].

New advances in dry preservation have been envisioned as a solution to overcome the challenges mentioned above, including for long-term storage of oocytes [2]. Advantages of dry preservation span from the simple handling and storage of the samples to the flexibility of transporting samples at ambient temperatures [2,6]. Dry preservation is based on creating a stable glass that can be stored without refrigeration and can circumvent issues that arise in typical cryopreservation to better enable field work and facilitate easier transportation and storage of genetic resources. A glass is formed when a substance is brought below its glass transition temperature (Tg), either by cooling or dehydration, without allowing crystallization to occur. A system below this temperature exhibits solid-like behavior but remains amorphous like its liquid form [7]. Therefore, trehalose is a preferred protectant for dry state storage, as it has a high glass transition temperature, enabling room temperature storage in the amorphous state under controlled humidity conditions. The Tg of trehalose delineates the transition between the stable glassy state and the supersaturated liquid state, which is highly prone to potentially damaging crystallization [8]. There are two main requirements for dry preservation with trehalose. The first is reaching the glassy, amorphous state by dehydration, and the second is the maintenance of this state with storage at a controlled humidity and temperature [9].

Previous studies using domestic cat cells as a model have shown that an immature oocyte at the germinal vesicle (GV) phase can be preserved and recovered reliably after preservation [10,11]. Taking advantage of those features, we have explored dry preservation of isolated GVs as an alternative to cryopreservation [12,13]. After oocyte membrane permeabilization with hemolysin, glass-forming disaccharide trehalose was incorporated into the oocyte to surround the GV. Removal of the water by microwave-assisted dehydration then led to attainment of an amorphous glassy state [9]. Our earlier studies also reported that, to maximize the recovery and DNA integrity of dry preserved samples, it was necessary to achieve and maintain a moisture content below 0.105 g H_2_O/g of dried weight (g DW) [9]. This ensured that samples remained in the glassy state at room temperature. However, at ambient temperatures (22–25 °C), trehalose will crystallize if exposed to a 43% relative humidity (RH) for more than few hours, by absorbing enough water (0.105 g H_2_O/g DW) to form trehalose dihydrate [9]. At a higher temperature, the likelihood of crystallization under these moisture conditions would increase even further due to the increased molecular mobility.

Because of potential issues related to the maintenance of tightly controlled temperatures and humidity levels during storage of dried products, the goal of the present study was to characterize the impact of additives that may help prevent DNA damage during short-term excursions from ideal storage conditions. It has been shown that the introduction of different salts to trehalose solutions can help prevent crystallization of the trehalose by binding to the available water or otherwise interfering with the crystallization process [14,15]. Therefore, choline acetate was chosen as additive to the preservation solution as the large choline cation provides steric hindrance to the system, preventing crystal formation by molecule alignment, and the ionic interactions between the acetate ion and water left water unavailable for trehalose crystallization [14]. It was also previously shown that choline salts in particular are highly effective at suppressing trehalose crystallization [14]. The choline salt was also selected due to its natural occurrence in cells and animals. Choline is a necessary nutrient in the body, as it contributes to the formation of the lipid bilayer of cell membranes [16].

Therefore, the objective of this study was to determine if the addition of choline acetate to trehalose solution could enable a wider range of storage conditions for preservation of nuclei from fully grown oocytes by allowing temporary humidity excursions (>44% relative humidity) that may lead to crystallization of trehalose and loss of DNA integrity.

## 2. Materials and Methods

### 2.1. Oocyte Collection

The procedure followed for this study was the same procedure outlined in our previous studies [9]. Briefly, oocytes were obtained from ovaries excised through routine ovariohysterectomies performed at a local veterinary clinic. Entire reproductive tracts, from uterine horn to ovary, were excised, then placed into a solution of 196 mL of phosphate-buffered saline (PBS) and 4 mL of a prepared solution of 10,000 units penicillin and 10 mg streptomycin/mL (Sigma-Aldrich, St. Louis, MO, USA) at 4 °C. The tracts were then transported at 4 °C to the laboratory within 2 h. The ovaries were dissected from the reproductive tract and were placed into a dish of handling medium comprised of HEPES-buffered minimum essential medium (MEM; Thermo Fisher Scientific, Waltham, MA, USA; HEPES; Sigma-Aldrich, MO, USA) supplemented with 2.0 mM L-glutamine, 1.0 mM sodium pyruvate, 100 IU/mL penicillin, 100 IU/mL streptomycin and 4 mg/mL bovine serum albumin (BSA; Sigma-Aldrich, St. Louis, MO, USA). Ovaries were then sliced under a stereo microscope to release cumulus-oocyte complexes (COCs) from the antral follicles. The COCs were isolated, then classified by the quality criteria described previously [12]. Grade 1 and 2 COCs were collected for use in this study. These are described as having a uniform, dark cytoplasm and at least 5 layers of compacted cumulus cells for grade 1, or fewer than 5 layers for grade 2. The COCs were denuded to remove the layers of cumulus cells surrounding the oocytes by incubation at 37 °C in a hyaluronidase solution, followed by a procedure of vortexing and washing thoroughly with handling medium.

### 2.2. Preparation and Exposure to Trehalose or Trehalose/Choline Acetate Solutions

Trehalose/choline acetate solutions were prepared with a 2:1 molar ratio in Tris-EDTA (TE; Sigma-Aldrich, St. Louis, MO, USA) buffer solution to yield 20% weight by volume (*w*/*v*) concentration (Table 1). Oocytes were first incubated in 10 µg/mL hemolysin (Sigma-Aldrich, St. Louis, MO, USA) for 15 min at 37 °C [12], then were exposed to 300 µL droplets of trehalose or trehalose/choline acetate solution for 10 min at room temperature to allow for solution diffusion across the membrane. After the incubation, oocytes were either washed three times in recovery medium (handling medium without BSA) for 10 min at room temperature to remove the trehalose/choline salt solution or processed for microwave-assisted drying (see below).

### 2.3. DNA Fragmentation and Chromatin Configuration Analysis

**Figure 1:**
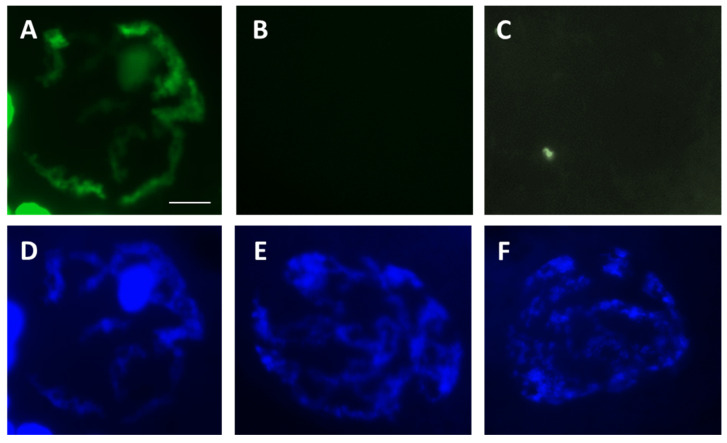
Representative images of germinal vesicles after TUNEL staining (**A**–**C**) and DAPI counterstaining (**D**–**F**). (**A**,**D**) positive control (DNase-treated). (**B**,**E**) negative control (TdT-omitted). (**C**,**F**) dried/rehydrated germinal vesicle positive to TUNEL. White scale bar = 10 µm for all pictures.

**Figure 2:**
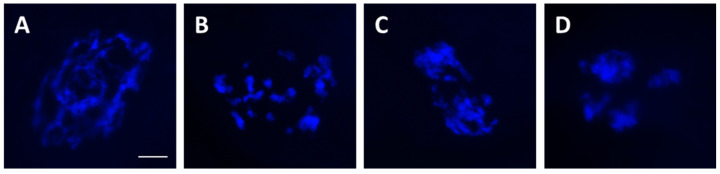
Representative images of germinal vesicles with normal (**A**) and different types of abnormal (**B**–**D**) chromatin configurations after drying and rehydration. Chromatin was stained with DAPI. White scale bar = 10 µm for all pictures.

Oocytes were placed in a droplet of 4% paraformaldehyde to be fixed for 8 h, then stored in individual tubes with 70% ethanol until ready for analysis. DNA fragmentation was determined by the terminal deoxynucleotidyl transferase dUTP nick end labeling (TUNEL) assay using an in situ cell death detection kit (Roche Applied Science, Indianapolis, IN, USA). After rinsing off ethanol with PBS, oocytes were permeabilized with 0.5% Triton X100 in PBS for 30 min at room temperature before exposure to TUNEL reaction mixture for 1 h at 38° C. Oocytes were then washed three times in PBS and mounted on slides with Vectashield containing DAPI (Vector Labs, Inc., Burlingame, CA, USA) to assess chromatin configuration. For TUNEL positive controls, oocytes were exposed to DNase for 10 min prior to TUNEL staining; for negative controls, TdT enzyme mix was omitted in the reaction mixture. Each slide was examined under an epifluorescence microscope (Olympus BX41; Olympus Corporation, Melville, NY, USA) using SPOT software 5.0 (Diagnostic Instruments, Inc., Sterling Heights, MI, USA). Oocytes with TUNEL staining or not recorded based on the presence/absence of green fluorescence in GVs [12] (Figure 1). DNA without detectable TUNEL staining was considered intact. Percentage of DNA integrity was calculated as number of oocytes without TUNEL staining over total number of oocytes in each treatment group. Normal GV chromatin had a reticular configuration [17] as determined by the DAPI counterstain. Any other chromatin configurations, such as large clumps, were categorized as abnormal (Figure 2). Percentage of chromatin abnormality was calculated as number of oocytes with abnormal chromatin over total number of oocytes in each treatment group.

### 2.4. Standard Drying Curves for Trehalose and Trehalose/Choline Acetate Solutions

Microwave-assisted drying curves were determined to identify suitable endpoints for dehydration studies. Anhydrous weights of each solution were obtained by following the protocol outlined in our previous studies [18]. Briefly, glass Petri dishes and fiberglass filters (Whatman, GE life Sciences, Marlborough, MA, USA) were stored in a desiccator for 24 h prior to use.

Triplicate samples of 400 µL of each preservation solution were placed on filter papers inside of glass dishes in a 125 °C oven. Samples were dried for a total of 96 h, with mass measurements every 24 h to establish sequential measurements with no change in mass. The percentage of solid content of the solution was then calculated and used as a reference in subsequent calculations.

To determine the drying kinetics of each solution, droplets of 40 µL volume were placed on the center of 0.5-inch diameter glass fiber filter paper. Using a ventilated carousel attachment, samples were dried in 5 min increments using a microwave (CEM SAM 255, Matthews, NC, USA) operating at 20% power. Humidity was controlled during the process at 11 +/− 1% RH by incorporating dry air into a sealed chamber, which was monitored with a hygrometer (HH314A, Omega, Norwalk, CT, USA), The sample moisture content was evaluated at every interval up to 40 min using a Karl Fischer volumetric titrator (V20S, Mettler Toledo, Columbus, OH, USA) with an attached microbalance (Mettler Toledo, Columbus, OH, USA). Measurements from Karl Fisher titration (wt% water) were converted to grams of water per gram of dry weight (g H_2_O/g DW) and then the average (±SD) was plotted as a function of drying time for each solution (n = 4). Within each time point, values were compared using Student’s *T*-test.

### 2.5. Microwave-Assisted Drying of GV Oocytes

After exposure to trehalose or trehalose/choline acetate solutions (see above), GV oocytes were deposited in 40 µL of solution, onto a dry filter paper disk, which had been pre-equilibrated in the 11% RH (±2%) controlled chamber at least 24 h in advance. Samples were placed onto the microwave carousel, together with a solution control used for Karl Fisher titration, and then exposed to microwave within the 11% RH humidity chamber. The samples were immediately dried with microwave assistance for 30 min. This duration had previously been determined to result in the attainment of equilibration moisture content in both solutions.

### 2.6. Sample Storage Conditions and Exposure to Adverse Conditions

After drying, filters containing GV oocytes were placed into glass vials, which had been preequilibrated to the environmental 11% RH air by placing them in the controlled humidity chamber at least 30 min prior to use. The glass vials were then sealed with a rubber stopper and banded with an aluminum strip that was crimped to the lip of the vial [9]. The vials were placed into a storage case on the lab bench. The samples were kept in this storage case for 2 weeks. After 2 weeks, samples were subjected to two different humidity levels. As a control, half of the samples—one of each prepared pair—were kept in their glass vials, assumed to be sealed correctly and thus maintained at 11% RH. This was considered ‘ideal conditions’, since other work had shown that under these conditions, dried GV oocytes to the appropriate moisture level can remain in a glassy state during storage up to 8 weeks [9]. The other half—the second of each pair—were opened and placed in an environmental chamber (Environmental Test Chamber 6010, Caron, Marietta, OH, USA) at 23 °C and 76% RH for 16 h. This level of humidity was selected because previous work has indicated that at this humidity, pure trehalose glass will rapidly crystallize, while the trehalose–choline acetate combination should resist crystallization [14]. Therefore, this treatment of an open vial at 76% RH was considered ‘adverse conditions’, subjecting samples to a humidity excursion.

### 2.7. Rehydration and Recovery

Samples were rehydrated and removed from their substrate (glass fiber filter) according to the method of Wang et al. [9]. Briefly, a droplet of recovery medium was slowly dropped onto the filter substrate. After 5 min, oocytes were recovered from the filter substrate. Oocytes that were on the surface of the filter or that had been released from the substrate and had been suspended in the recovery medium were collected. After an initial search, if not all oocytes had been recovered, the filters were left to rehydrate for 30 min. Any additional oocytes were recovered. The recovery rate was defined as the ratio of oocytes recovered from the filter papers and used for further testing compared to the initial number placed into the treatment groups.

### 2.8. Raman Analysis

This analysis was performed to confirm that the glassy state was achieved following microwave processing. Raman spectroscopy is a non-destructive analysis technique that uses a high energy excitation laser to probe the vibrational modes of the molecules of a substance [19,20]. When the molecules are in an amorphous state and there is little to no order in the system, the movement of the molecules is slow and large, with the entire molecule vibrating. When in a crystalline conformation, and the molecules are stacked and ordered, the vibrations are small and minor. This allows the spectrometer to pick up the vibrations of individual atoms within the molecule, rather than those of the entire molecule. In this way, Raman spectrum differences between amorphous and crystalline structures can be observed, thereby confirming the expected state of the materials following dehydration and storage [21].

Raman analysis was performed according to the method of Wang et al. [9]. The measurements were conducted with a Horiba Xplora confocal Raman microscope with a 1200 g/mm grating. The excitation laser had a wavelength of 785 nm. Data were taken using a 40× microscope lens. The laser power was measured at ~15 mW at full power. Hole size was set at 100 µm. All measurements were performed at room temperature. The acquisition time of each Raman spectrum was 10 s.

To create a glassy trehalose matrix, droplets of 40 µL were placed on fused quartz microscope cover slides (Alfa-Aesar, Tewksbury, MA, USA) and placed into a 120 °C oven to completely dry the sample. The amorphous sample was scanned with a Raman microscope to provide an amorphous control spectrum, then placed into a 76% RH environmental chamber for approximately 8 h until crystals had visibly formed in the material. The sample was again scanned, yielding the crystallized trehalose control spectra [21]. Amorphous trehalose/choline acetate control was created by microwaving a droplet of trehalose choline acetate solution on a fused quartz cover slip until the sample had become solid. Crystallized trehalose/choline acetate control was obtained by microwaving the droplet until a viscous skin formed followed by 60 min holding at 25 °C and 55% RH until crystals formed in the composition.

### 2.9. Experimental Design and Statistical Analysis

A preliminary experiment was conducted to determine if the addition of choline acetate to the traditional trehalose based drying medium had any inherent toxicity effect, oocytes were first exposed to the trehalose/choline acetate solution without a drying step. Oocytes (n = 93 in 5 replicates) were randomly assigned to either fresh control or trehalose/choline acetate-exposed group. Samples in the latter group were exposed to trehalose/choline acetate, recovered, and subjected to DNA fragmentation and chromatin configuration analysis as described above. Data were analyzed by paired *t*-test.

To examine the effect of choline acetate additives on DNA integrity and chromatin configuration under regular and adverse storage conditions, oocytes (n = 242 in 9 replicates) were randomly assigned to treatment groups (trehalose control and trehalose/choline acetate) in each replicate. Each treatment group was further divided into two processing groups (storage at 11%RH or with short-term exposure at 76% RH) before recovery. Statistical analysis was conducted with standard analysis of variances (ANOVA) with a standard *p* value of 0.05. The software SPSS (SPSS 28, IBM, Armonk, New York, NY, USA) was used for performing comparisons among samples.

To differentiate between amorphous and crystallized samples, Raman spectroscopy was used. Samples were created and exposed to ideal and adverse relative humidity conditions in a manner that paralleled the previous experiments (3 samples per condition and 3 scans per sample, from different areas of the sample). Control spectra of amorphous and crystalline compositions were as described above for comparison.

## 3. Results

### 3.1. Effect of Choline Acetate Addition to Trehalose Solution on DNA Integrity and Chromatin Configuration before Drying

A preliminary study was performed to evaluate possible toxic effects of the choline acetate on DNA and chromatin configuration. There were no differences in DNA integrity between treatment group and controls (*p* > 0.05), with little to no DNA fragmentations (ranging from 86 to 100%). However, incidence of abnormal chromatin configurations was higher (*p* < 0.05) when oocytes were exposed to choline salts and trehalose solution (64.5 ± 17.0%) compared to trehalose solution alone (36.6 ± 29.8%).

### 3.2. Effect of Choline Acetate Addition to Trehalose Solution on Drying Curves

**Figure 3:**
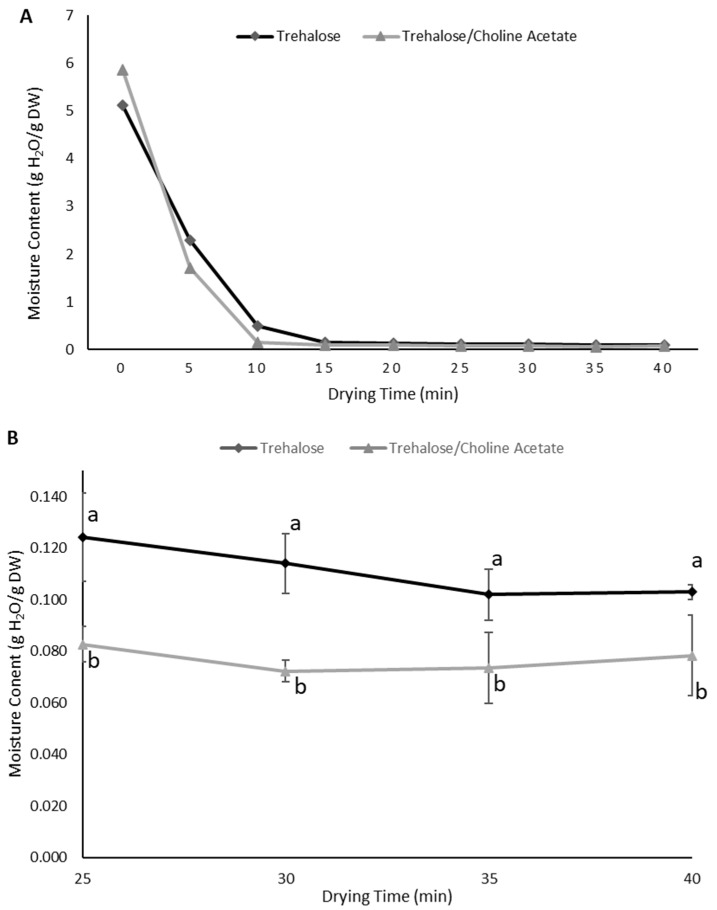
Moisture content as a function of drying time with microwave assistance (in 11% RH environment) with or without choline acetate addition. (**A**) Entire drying curve from 0 to 40 min. (**B**) Details on later drying times only (mean ± SD). ^a,b^ Within each time point, values with different superscripts are different (*p* < 0.05).

The weight of the solutes in the Tris-EDTA buffer were considered insignificant and did not impact the weight of solids in the solutions. A rapid decrease in moisture content was observed between 0 and 15 min of drying (Figure 3A). Between 15 and 40 min, decrease in moisture content was slower than in the previous interval (Figure 3A). There were no more decreases in moisture content after 25 min of drying (Figure 3B). Therefore, a drying time of 30 min was chosen for the next experiments. However, the end moisture content of the trehalose–choline acetate solution was significantly lower than the trehalose solution with no salt additive (*p* < 0.05; Figure 3B).

### 3.3. Effect of Choline Acetate Addition to Trehalose Solution on Oocyte Recovery, DNA Integrity, and Chromatin Configuration after Drying and Storage

**Figure 4:**
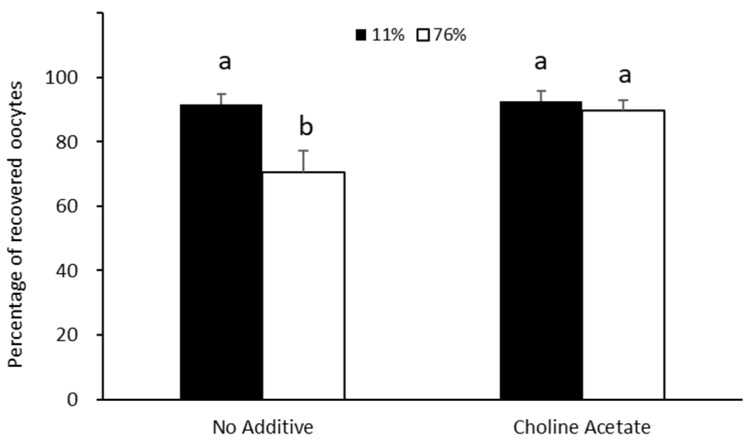
Effect of choline salt addition to trehalose solution during drying and storage on recovery of germinal vesicles in ideal (11% RH) and adverse conditions (76% RH). Values are expressed as average ± SEM. ^a,b^ Bars with different letters differ significantly *p* < 0.05).

Samples were exposed to treatments of either ideal (11% RH) or adverse (76% RH) conditions for 16 h after drying and storage at 11% RH. In the control trehalose solution, under ideal conditions (11% RH), the recovery rate was 91.6% (Figure 4). However, the recovery of samples stored in trehalose in adverse conditions at 76% RH (70.6%) was less successful (*p* < 0.05) than those under ideal conditions (11% RH) (Figure 4). Samples in the preservation solution with choline acetate had higher (*p* < 0.05) recovery success when compared to the trehalose solution alone under adverse conditions (76% RH). Furthermore, recovery success was high and not different (*p* > 0.05) between the conditions (11% and 76% RH) when choline acetate was added to the trehalose solution (Figure 4).

**Figure 5:**
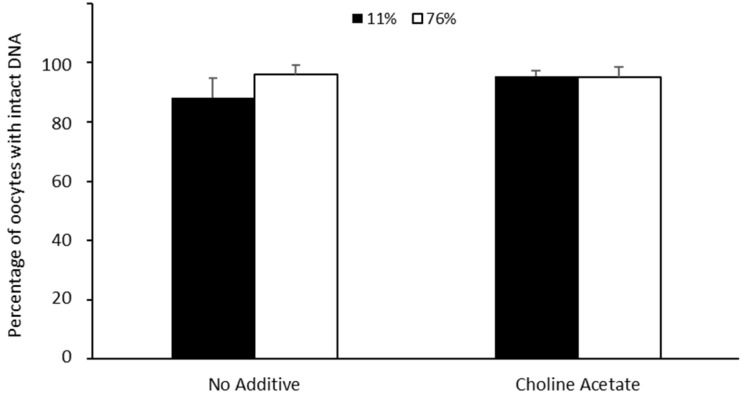
Effect of choline salt addition to trehalose solution during drying and storage on DNA fragmentation in ideal (11% RH) and adverse conditions (76% RH). Values are expressed as average ± SEM. There were no significant differences (*p* > 0.05) between treatment groups.

All samples exhibited high levels of DNA integrity (ranging from 88 to 96%; Figure 5). No differences (*p* > 0.05) were found either between solutions or within solutions under different conditions (Figure 5).

**Figure 6:**
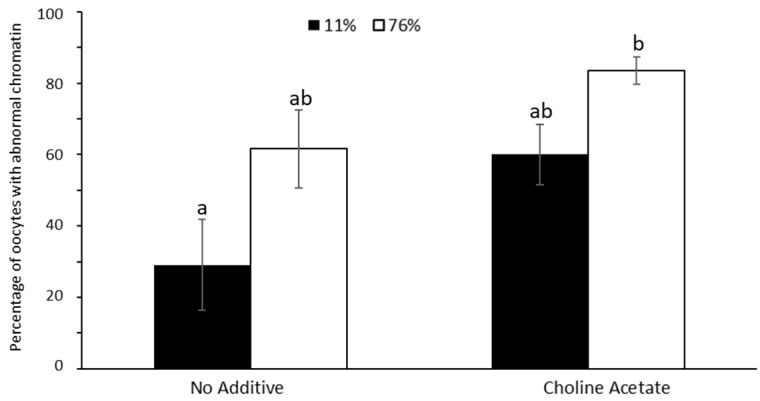
Effect of choline salt addition to trehalose solution during drying and storage on chromatin configuration in ideal (11% RH) and adverse conditions (76% RH). Values are expressed as average ± SEM. ^a,b^ Bars with different letters differ significantly *p* < 0.05).

Regarding the chromatin configuration, adverse conditions (76% RH) tended to increase the incidence of abnormalities with the trehalose solution alone and with addition of choline acetate (Figure 6). Compared to trehalose solution alone at 11% RH, choline salt addition at 76% RH led to a higher percentage (*p* < 0.05) of abnormal chromatin.

### 3.4. Effect of Choline Acetate Addition to Trehalose Solution on Crystallization during and after Drying

**Figure 7:**
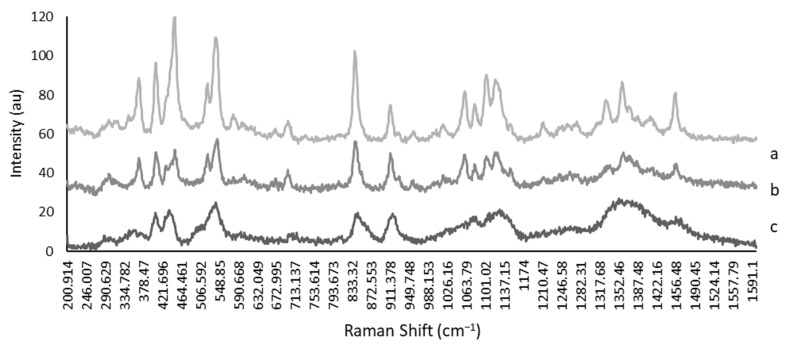
Raman spectra of trehalose solution. Sharper and more numerable peaks are associated with a crystalline conformation (a), compared to broadened peaks in the amorphous sample (c). The spectrum for samples held at 76% RH is given in (b). Samples stored at this condition are consistent with crystalline samples.

Differences between amorphous and crystalline conformations of trehalose only (control) were observed in the shapes of the spectra (Figure 7). Peaks were broadened and less distinct in the amorphous sample (c), compared to the crystalline sample (a) (Figure 7). Samples exposed to the adverse conditions of 76% RH (b) contained the same sharp and distinct peaks as the crystalline control sample, indicating that it was in fact crystalline following an adverse conditions challenge (Figure 7).

**Figure 8:**
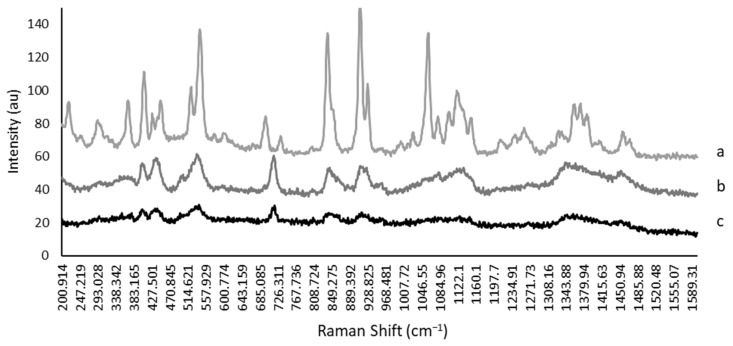
Raman spectra of trehalose solution with choline acetate. Sharper and more numerable peaks are associated with a crystalline conformation (a), compared to broadened peaks in the amorphous sample (c). The spectrum for samples held at 76% RH is given in (b). Samples stored at this condition are consistent with amorphous samples.

The Raman spectra for trehalose–choline acetate combination are shown in Figure 8. Peaks in the crystalline sample (a) were much more intense and sharper than in the amorphous sample (c). Based on the spectra, samples that were subjected to the adverse environmental conditions (b) (76% RH) remained in an amorphous state with no evidence that crystals had been formed (Figure 8).

## 4. Discussion

The primary reason for adding choline salt to trehalose was to address potential issues related to long-term storage of dried samples, specifically, to suppress crystallization during excursions from ideal humidity conditions (increase from 11% to 76% of RH). Compared to trehalose exposure alone, the addition of a choline acetate to trehalose enabled to reach lower moisture contents after 25 min of microwave-assisted drying. Sample recovery after drying and storage also was better in the presence of choline acetate. The integrity of DNA in the germinal vesicles was not affected by the presence of choline acetate. However, exposure to choline salt had a negative effect on the chromatin configuration. For the first time, we also demonstrated that choline acetate addition helped to maintain an amorphous state and better preserve oocyte’s nuclei during adverse experimental conditions (increase in RH).

Preliminary data demonstrated that there was no toxic effect of choline acetate exposure on the oocyte’s DNA. This is similar to previous observations. Salts are very hygroscopic [14]. The water binding nature of the salts is hypothesized to keep water unavailable for the formation of trehalose dihydrate, and thus may prevent oocyte’s nuclei from a localized crystallization event. We then observed that choline acetate addition to trehalose solution enabled to reach lower moisture contents after 25 min of microwave-assisted drying, which confirmed the properties of the salt. Importantly, the osmotic pressure that these solutions would exert on intact cell membranes was not relevant in our experimental conditions, because the oocyte membranes were permeabilized (with hemolysin) as part of the preservation processing. The incidence of abnormal chromatin during choline salt exposure before dehydration (64.5%) was similar after dehydration and storage in ideal conditions (11% RH; Figure 6); however, it was multiplied by 1.5 after drying and storage at 76%RH. Even though the types of abnormal chromatin configurations were not different before and after dehydration, the negative effect of choline salt addition on the chromatin configuration requires further studies to mitigate the issue.

A drying time of 30 min was selected as the appropriate amount of time for the study. Our previous studies had determined, by use of one-way temperature sensors, that thermal processing of 30 min did not exceed natural physiological temperatures, therefore it was unlikely to cause thermal damage to the cells [9].

In a previous study, oocytes dried in trehalose and stored at 11% yielded a recovery rate of 72.7 ± 12.8% after 8 weeks of storage [9]. In the control trehalose solution of the present study, under ideal conditions (11% RH), the recovery rate was 91.6 ± 9.7%. This improvement could be attributed to a more thorough and defined protocol for recovery that was developed for the current study. Sample recovery was better in the presence of choline acetate, regardless of the excursion from 11 to 76% RH. This may be attributed to the avoidance of crystallization by the addition of choline acetate. The hygroscopic nature of the salt was also hypothesized to improve the recovery, because a rapid absorption of water might aid in the dissolution of the trehalose glass and therefore increase the likelihood of recovery of the oocytes from the filter paper used for treatment and storage [22].

In a former report, it was observed that trehalose crystallizes under humidity conditions that exceed 44% RH [15]. Therefore, we had hypothesized that crystal formation under adverse humidity conditions could cause physical damage to the oocyte’s nuclei. Results of the TUNEL assays were more encompassing of the true effects of the treatments and preservation compositions. Consistent with prior research, there was no change in percentages of nuclei with TUNEL staining following dry preservation using trehalose-based compositions, indicating the novel compositions are effective even under ideal conditions [9]. Interestingly, Figure 5 showed no significant difference in DNA integrity between treatment groups and also between storage conditions. We could also conclude that crystallization caused by higher relative humidity during storage did not affect DNA integrity. Proportions of abnormal chromatin varied within conditions, so differences were not easy to detect between treatments. However, results were consistent with previous observations [6,12]. Overall, dehydration led to a lower incidence of DNA fragmentation and less structural damages of the chromatin than in vitrified oocytes [6,23].

Choline acetate addition helped to maintain an amorphous state during adverse experimental conditions. Adding salts to sugar systems has been shown to delay the onset of crystallization and can preserve the amorphous state of sugar systems under a wider range of environmental conditions [15,24,25,26,27]. Numerous studies have focused on the effect of anions on the stability of amorphous sugar compositions. For example, it has been reported that anions with hydrogen bonding ability can crosslink with sugar and increase the T_g_ of the mixture, delaying crystallization of the amorphous system [26]. As summarized by Ziaei [14], salts with large ions (like choline) can also delay crystallization via steric hindrance [14,25]. Water-ion interactions that prevent the formation of trehalose dihydrate are another potential stabilizing mechanism. Ziaei studied the effect of a variety of different ions on the crystallization tendency of trehalose and observed considerable reduction in the time to crystallization when choline acetate was added to trehalose. This provided the basis for the current study [14]. The salt was combined with trehalose in a 20% weight by volume concentration, which is below the saturation points of the solutes, thus preventing spontaneous crystallization during handling. Although starting with dilute solutions requires a longer drying time than solutions that are closer to the saturation limit, the low viscosity made the solutions easier to transfer during the study, potentially reducing shear stress on oocyte’s nuclei during handling procedures.

## 5. Conclusions

Choline salt addition to trehalose solution enabled us to reach a lower moisture content during microwave-assisted dehydration. It also allowed a better recovery of the oocyte’s nuclei. While DNA integrity was not affected by the different treatments, the chromatin configuration was damaged in the presence of choline salts when storage occurred in adverse conditions. Although ideal storage methods can be maintained in environmental chambers with fixed relative humidity and temperature, during shipping, these conditions are difficult to maintain. There is always the risk of damage to packaging, shipping delays, as well as temperature and relative humidity excursions. The present study developed more robust preservation solutions that do not crystallize in response to adverse conditions, providing protection against unexpected excursions that occur as a part of damage or human error. This information will also be useful to future users addressing the pressing needs to increase simple and cost-effective storage of germplasms for the long-term.

## Figures and Tables

**Table 1:** Compositions of preservation solutions. Trehalose and trehalose/choline acetate solutions were made by combining calculated amounts of solutes and adding tris-EDTA buffer to make 25 mL stock solutions of 20% (*w*/*v*) concentration. Trehalose was calculated as an anhydrous molecule (342.496 g/mol) and purchased from supplier as a trehalose dihydrate (378.33 g/mol).

Preservation Solution	Trehalose (mol)	Trehalose (g)	Choline Acetate (mol)	Choline Acetate (g)	Wt%
Trehalose (Control)	0.014599	5.5231	0.0	0.0	20
Trehalose/Choline acetate	0.0117	4.4604	0.0059	0.9629	20

## Data Availability

All research data are shared in the manuscript. If needed, raw data can be requested from the corresponding authors.
